# SREBP1-Induced Glutamine Synthetase Triggers a Feedforward Loop to Upregulate SREBP1 through Sp1 O-GlcNAcylation and Augments Lipid Droplet Formation in Cancer Cells

**DOI:** 10.3390/ijms22189814

**Published:** 2021-09-10

**Authors:** Jin-Wei Jhu, Jia-Bao Yan, Zou-Han Lin, Shih-Chieh Lin, I-Chen Peng

**Affiliations:** 1 Department of Life Sciences, National Cheng Kung University, Tainan City 701, Taiwan; q976583460@gmail.com (J.-W.J.); oscar845943@gmail.com (J.-B.Y.); a60260211@gmail.com (Z.-H.L.); 2 Institute of Basic Medical Sciences, College of Medicine, National Cheng Kung University, Tainan City 701, Taiwan; Jaylin@mail.ncku.edu.tw

**Keywords:** sterol regulatory element-binding protein 1, glutamine synthetase, O-GlcNAcylation, lipid droplet, cancer

## Abstract

Glutamine and lipids are two important components of proliferating cancer cells. Studies have demonstrated that glutamine synthetase (GS) boosts glutamine-dependent anabolic processes for nucleotide and protein synthesis, but the role of GS in regulating lipogenesis remains unclear. This study identified that insulin and glutamine deprivation activated the lipogenic transcription factor sterol regulatory element-binding protein 1 (SREBP1) that bound to the GS promoter and increased its transcription. Notably, GS enhanced the O-linked *N*-acetylglucosaminylation (O-GlcNAcylation) of the specificity protein 1 (Sp1) that induced SREBP1/acetyl-CoA carboxylase 1 (ACC1) expression resulting in lipid droplet (LD) accumulation upon insulin treatment. Moreover, glutamine deprivation induced LD formation through GS-mediated O-GlcNAc-Sp1/SREBP1/ACC1 signaling and supported cell survival. These findings demonstrate that insulin and glutamine deprivation induces SREBP1 that transcriptionally activates GS, resulting in Sp1 O-GlcNAcylation. Subsequently, O-GlcNAc-Sp1 transcriptionally upregulates the expression of SREBP1, resulting in a feedforward loop that increases lipogenesis and LD formation in liver and breast cancer cells.

## 1. Introduction

Sterol regulatory element-binding protein 1 (SREBP1) is the major transcription factor that regulates the expression of lipogenic genes [1]. De novo lipogenesis is controlled by acetyl-CoA carboxylase 1 (ACC1), which is regulated at the transcriptional and post-translational levels [2,3]. Transcriptionally, insulin induces SREBP1 binding to the ACC1 promoter resulting in ACC1 transactivation and augmented lipogenesis [1,3]. In addition to lipogenic genes, SREBP1 is predicted to regulate various non-lipogenic genes [4,5] and plays an important role in autophagy [4], pro-fibrotic signaling [6], endoplasmic reticulum stress [7], and metabolic circadian rhythm [8]. However, the function of SREBP1 for regulating glutamine metabolism remains unknown.

Glutamine functions as a nitrogen donor, a signaling molecule, and an exchanger used to import other amino acids in proliferating cancer cells [9]. Many cancer cells rely on extracellular glutamine uptake and cannot survive in glutamine-deprived conditions. However, some cancer cells that express high levels of glutamine synthetase (GS), the enzyme responsible for de novo glutamine production, have increased glutamine-dependent anabolic processes and are more resistant to glutamine deprivation than cells with low GS expression [10,11,12,13,14,15]. Additionally, glutamine is essential for de novo uridine-diphosphate-N-acetylglucosamine (UDP-GlcNAc) biosynthesis and protein O-linked *N*-acetylglucosaminylation (O-GlcNAcylation) [16,17]. Protein O-GlcNAcylation is involved in the regulation of diverse cellular processes including transcription, epigenetic modifications and cell signaling [18]. The O-GlcNAcylation of the transcription factor specificity protein 1 (Sp1) is required for binding to the promoter of and inducing the expression of SREBP1 [19,20,21]. Although adding glutamine in the media increases Sp1-induced SREBP1 expression [22], the role of GS on SREBP1 induction via O-GlcNAcylation remains unclear.

In this study, we demonstrate that SREBP1 binds to the promoter, and induces the expression of GS resulting in lipid droplet (LD) formation through O-GlcNAc-Sp1/SREBP1/ACC1 signaling in liver and breast cancer cells. These findings reveal the expanded function of SREBP1 for regulating glutamine metabolism and reveal the effects of GS on protein O-GlcNAcylation and lipogenesis in cancer.

## 2. Results

### 2.1. Insulin and Glutamine Deprivation Upregulate GS Expression through SREBP1

**Figure 1:**
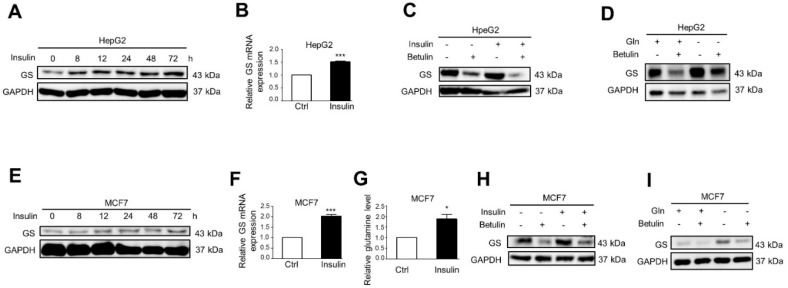
Insulin and glutamine deprivation upregulate GS expression through SREBP1. (**A**,**B**) HepG2 cells were treated with 200 nM insulin for indicated time periods (**A**) or with 200 nM insulin for 48 h (**B**). (**C**) HepG2 cells were treated with 50 µM SREBP1 inhibitor betulin for 30 min prior to 200 nM insulin treatment for 48 h. (**D**) HepG2 cells were treated with 50 µM SREBP1 inhibitor betulin for 30 min prior to glutamine deprivation for 24 h. (**E**,**F**) MCF7 cells were treated with 200 nM insulin for indicated time periods (**E**) or with 200 nM insulin for 48 h (**F**). (**G**) MCF7 cells were treated with 200 nM insulin for 72 h followed by intracellular glutamine quantification. (**H**) MCF7 cells were treated with 50 µM SREBP1 inhibitor betulin for 30 min prior to 200 nM insulin treatment for 48 h. (**I**) MCF7 cells were treated with 50 µM SREBP1 inhibitor betulin for 30 min prior to glutamine deprivation for 24 h. Cell lysates were analyzed by Western blot with specified antibodies or qRT-PCR with specific primers. Data represent means plus SEM. * *p* < 0.05 and *** *p* < 0.001.

We have previously reported the lipogenic effect of insulin on SREBP1 induction and cell growth in liver and breast cancer cells [3]. 200 nM insulin has been reported to mimic hyperinsulinemia in individuals with type 2 diabetes mellitus [3]. As glutamine is involved in cancer cell proliferation, we also tested the effect of insulin on GS induction. Indeed, GS protein and mRNA expression were induced by 200 nM of insulin in HepG2 (Figure 1A,B) or MCF7 (Figure 1E,F) cells which were attenuated by 50 µM of betulin, an SREBP1 inhibitor, in HepG2 (Figure 1C) or MCF7 (Figure 1H) cells. Consistent with previous results, GS expression was also induced upon glutamine deprivation [11,12,13] which was attenuated with 50 µM betulin in HepG2 (Figure 1D) or MCF7 cells (Figure 1I). In addition to GS induction, insulin also increased the level of intracellular glutamine in MCF7 cells (Figure 1G). These findings demonstrate that insulin and glutamine deprivation-induced GS expression is mediated by SREBP1.

### 2.2. SREBP1 Binds to the GS Promoter Leading to GS Transactivation

**Figure 2:**
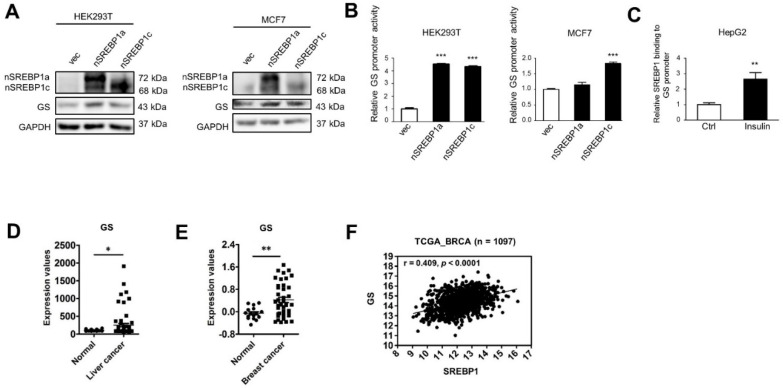
SREBP1 binds to the GS promoter leading to GS transactivation. (**A**) HEK293T and MCF7 cells were transiently transfected with vector, pcDNA3-nSREBP1a or pcDNA3-nSREBP1c (n: nuclear form) for 24 h and analyzed by Western blot with specified antibodies. nSREBP1: nuclear SREBP1. (**B**) HEK293T and MCF7 cells were transiently transfected with vector, pcDNA3-nSREBP1a or pcDNA3-nSREBP1c along with the GS promoter conjugated firefly luciferase reporters and a Renilla luciferase internal control plasmid. Luciferase activities were quantified after 24 h, and promoter activities were calculated based on the ratios of firefly luminescence versus Renilla luminescence. Promoter activities of nSREBP1a or nSREBP1c-transfected cells were normalized to that of vector-transfected control cells as relative promoter activities (means plus SEM). *** *p* < 0.001 (ANOVA) represents the statistical significance compared to vector-transfected control cells. (**C**) The relative amount of SREBP1 binding to the GS promoter upon 200 nM insulin treatment for 48 h was analyzed by ChIP assay using the SREBP1 antibody followed by qPCR with specific primers in HepG2 cells. Fold enhancement represented the abundance of enriched DNA fragments over an IgG control and the number was shown as the mean plus SEM. ** *p* < 0.01. (**D**,**E**) GS expression values were shown in (**D**) liver (GSE87630) and (**E**) breast (GSE83591) cancer tissues compared to adjacent non-cancerous tissues (normal) from database analysis. * *p* < 0.05 and ** *p* < 0.01. (**F**) Expression levels of SREBP1 and GS in TCGA dataset of breast cancer were used to perform Pearson’s correlation analysis.

As members of the bHLHLZ family of DNA binding proteins, SREBP1 dimerizes and recognizes the sterol regulatory element (SRE) sequence ATCACCCCAC with permissive diversities in a few bases [23]. An analysis of the putative GS promoter regions using the Regulatory Sequence Analysis Tools and the indicated SREBF1 motif (MAO595.1) from JASPAR identified a SREBP1 binding sequence ATCACCTCAG (−1393 to −1384) in the human GS promoter. This result suggested GS may be transcriptionally regulated by SREBP1. The overexpression of nSREBP1a or nSREBP1c enhanced GS protein expression in HEK293T or MCF7 cells (Figure 2A). A luciferase assay indicated that nSREBP1a or nSREBP1c increased GS promoter activity in HEK293T or MCF7 cells (Figure 2B). These results corresponded to increased SREBP1 binding to the GS promoter in insulin-treated HepG2 cells (Figure 2C). Subsequently, we investigated whether these effects occur in human cancer samples. GS mRNA expression levels (Figure 2D,E) and SREBP1 mRNA expression levels [3] were higher in breast and liver cancer tissues compared to adjacent non-cancerous tissues according to GEO database analyses. Furthermore, a positive correlation was observed between SREBP1 and GS mRNA expression in breast cancer compared to adjacent non-cancerous tissues (r = 0.409, *p* < 0.0001) (Figure 2F). These results demonstrate that SREBP1 induces GS transactivation.

### 2.3. GS Triggers a Feedforward Loop of O-GlcNAc-Sp1/SREBP1/ACC1 Signaling That Induces LD Formation upon Insulin Treatment

**Figure 3:**
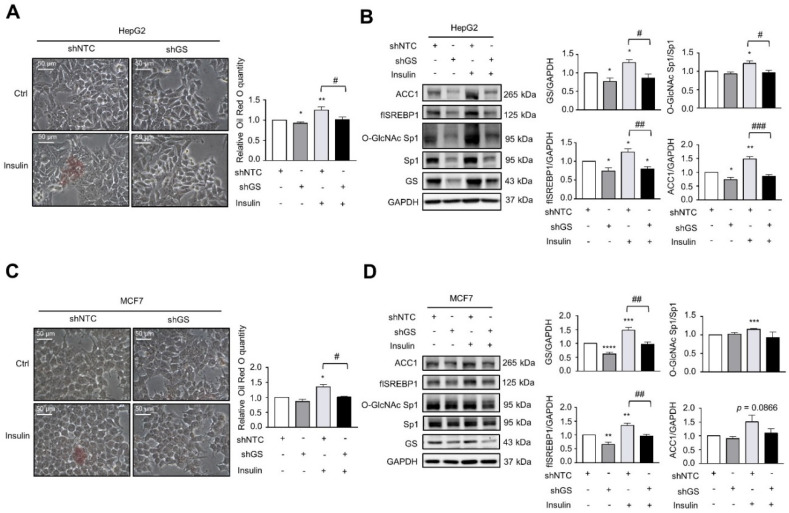
Knockdown of GS attenuates insulin-induced LD formation and O-GlcNAc-Sp1/SREBP1/ACC1 expression. (**A**,**B**) HepG2 cells stably transfected with non-targeting control or GS shRNA were cultured in the absence or presence of 200 nM insulin for 48 h. (**C**,**D**) MCF7 cells stably transfected with non-targeting control or GS shRNA were cultured in the absence or presence of 200 nM insulin for 48 h. (**A**,**C**) LD was stained with Oil Red O and normalized to protein concentrations for the quantitative analysis. (**B**,**D**) Cell lysates were analyzed by Western blot with specified antibodies. flSREBP1: full-length SREBP1. The bar graphs on the right represent the densitometry analyses of the ratios of indicated proteins. Data represent means plus SEM. * *p* < 0.05, ** *p* < 0.01, *** *p* < 0.001, and **** *p* < 0.0001 compared to control cells. ^#^ *p* < 0.05, ^##^ *p* < 0.01, and ^###^ *p* < 0.001 between indicated groups.

**Figure 4:**
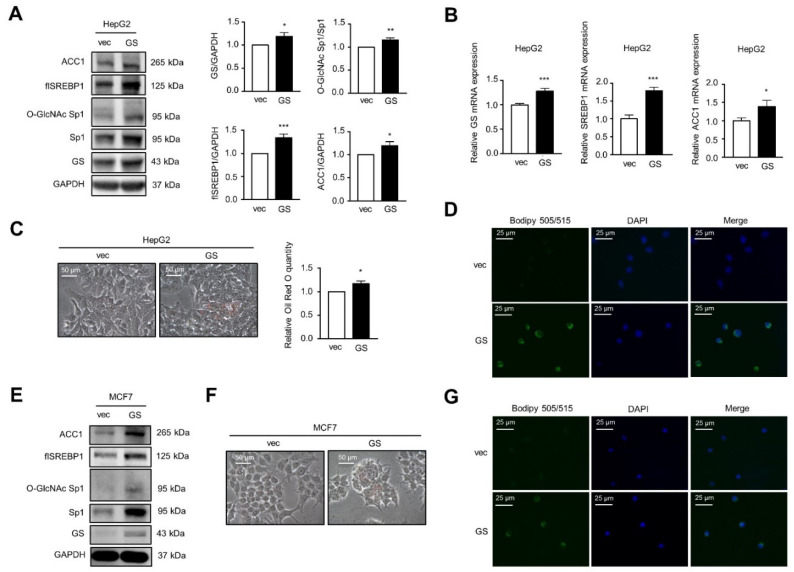
The overexpression of GS increases O-GlcNAc-Sp1/SREBP1/ACC1 expression and LD formation. (**A**–**D**) HepG2 cells stably transfected with vector or pBabe-GS were cultured. (**E**–**G**) MCF7 cells stably transfected with vector or pBabe-GS were cultured. (**A**,**E**) Cell lysates were analyzed by Western blot with specified antibodies. flSREBP1: full-length SREBP1. The bar graphs on the right represent the densitometry analyses of the ratios of indicated proteins. (**B**) Relative transcript levels were determined via qRT-PCR. (**C**,**F**) LD was stained with Oil Red O and normalized to protein concentrations for the quantitative analysis. Data represent means plus SEM. * *p* < 0.05, ** *p* < 0.01, and *** *p* < 0.001. (**D**,**G**) LD was stained with the BODIPY 505/515 (green) and the nuclei were stained with DAPI (blue).

Next, the biological function of insulin-induced GS was investigated. Insulin induced LD formation, whereas knockdown or inhibition of GS by 200 µM MSO reduced 200 nM insulin-induced LD formation in HepG2 cells (Figure 3A and Figure S1A). Intriguingly, knockdown of GS attenuated insulin-induced SREBP1/ACC1 protein expression (Figure 3B). Since glutamine is the precursor of UDP-GlcNAc for protein O-GlcNAcylation and SREBP1 expression is regulated by O-GlcNAc-Sp1 [22], we found that knockdown of GS decreased the level of O-GlcNAc-Sp1, which corresponded to reduced SREBP1 protein expression with or without insulin treatment (Figure 3B). Knockdown of GS had similar effects on insulin-induced LD formation and on O-GlcNAc-Sp1/SREBP1/ACC1 protein expression in MCF7 cells (Figure 3C,D). Furthermore, the overexpression of GS induced SREBP1/ACC1 protein and mRNA expression in HepG2 cells (Figure 4A,B). The overexpression of GS also increased the level of O-GlcNAc-Sp1 (Figure 4A), which was correlated with enhanced SREBP1 protein levels and the induction of LD formation in HepG2 cells (Figure 4C,D). Increased O-GlcNAc-Sp1/SREBP1/ACC1 protein expression (Figure 4E) and LD formation (Figure 4F,G) were also observed in MCF7 cells overexpressing GS. These findings indicate that GS increases Sp1 O-GlcNAcylation resulting in the induction of SREBP1/ACC1 expression and insulin-induced LD formation.

### 2.4. Glutamine Deprivation Induces LD Formation through GS-Mediated O-GlcNAc-Sp1/SREBP1/ACC1 Signaling, and GS Promotes Cell Survival under Glutamine Deprivation

**Figure 5:**
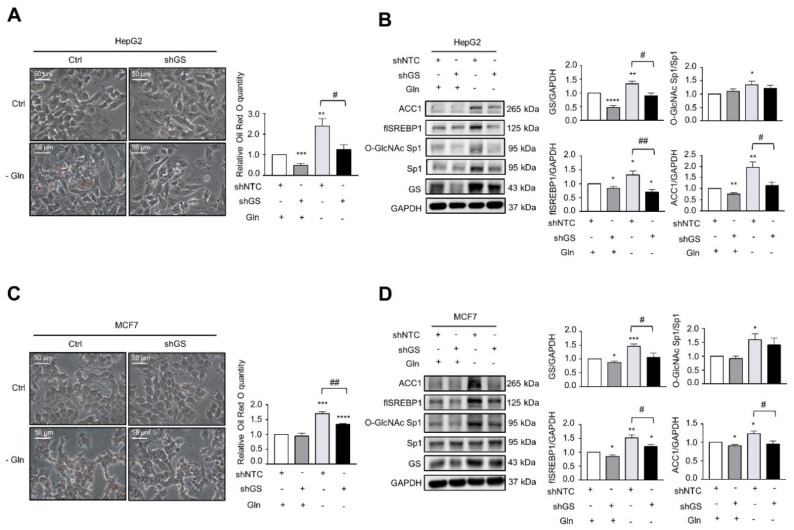
Knockdown of GS attenuates glutamine deprivation-induced LD formation and O-GlcNAc-Sp1/SREBP1/ACC1 expression. (**A**,**B**) HepG2 cells stably transfected with non-targeting control or GS shRNA were cultured under glutamine deprivation (-Gln) for 24 h. (**C**,**D**) MCF7 cells stably transfected with non-targeting control or GS shRNA were cultured under glutamine deprivation (-Gln) for 24 h. (**A**,**C**) LD was stained with Oil Red O and normalized to protein concentrations for the quantitative analysis. (**B**,**D**) Cell lysates were analyzed by Western blot with specified antibodies. flSREBP1: full-length SREBP1. The bar graphs on the right represent the densitometry analyses of the ratios of indicated proteins. Data represent means plus SEM. * *p* < 0.05, ** *p* < 0.01, *** *p* < 0.001, and **** *p* < 0.0001 compared to control cells. ^#^ *p* < 0.05 and ^##^ *p* < 0.01 between indicated groups.

In addition to insulin, and consistent with previous findings, we found that glutamine deprivation induced LD formation in HepG2 and MCF7 cells (Figure 5A,C) [24]. Knockdown or inhibition of GS by 200 µM MSO decreased glutamine deprivation-mediated LD formation in HepG2 (Figure 5A and Figure S1B) or MCF7 (Figure 5C) cells. Furthermore, glutamine deprivation induced GS, O-GlcNAc-Sp1, SREBP1, and ACC1 protein expression, which was attenuated if GS was knocked down in HepG2 (Figure 5B) or MCF7 (Figure 5D) cells. Moreover, the addition of 4 or 40 mM glutamine to glutamine-deprived HepG2 and MCF7 cells decreased glutamine deprivation-induced LD formation and GS protein expression (Figure S2A,B). In summary, these results demonstrate that GS mediates glutamine deprivation-induced LD formation through O-GlcNAc-Sp1/SREBP1/ACC1 signaling.

**Figure 6:**
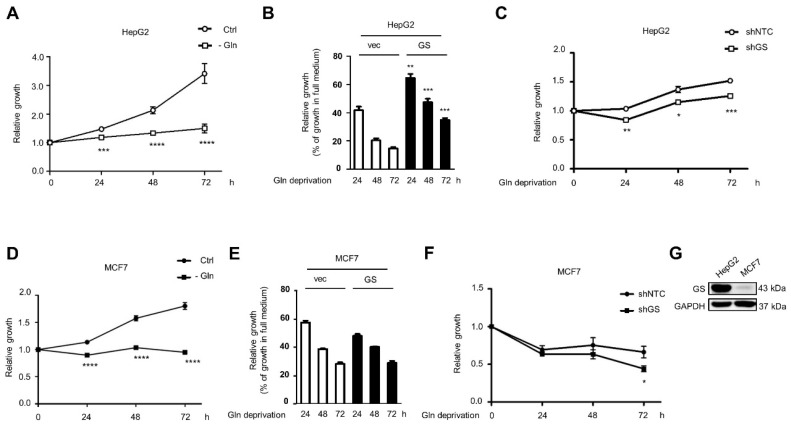
GS supports cell survival under glutamine deprivation. (**A**,**D**) Cell growth of HepG2 (**A**) and MCF7 (**D**) cells in full (Ctrl) and glutamine deprivation (-Gln) medium was measured by MTT assay and shown as the mean plus SEM. (**B**,**E**) HepG2 (**B**) and MCF7 (**E**) cells were stably transfected with vector control or pBabe-GS. Cell growth in full or glutamine (Gln) deprivation medium was measured by MTT assay. Relative growth in Gln deprivation medium (% of growth in full medium) was shown as the mean plus SEM. (**C**,**F**) HepG2 (**C**) and MCF7 (**F**) cells were stably transfected with non-targeting control or GS shRNA. Cell growth in Gln deprivation medium was measured by MTT assay and shown as the mean plus SEM. (**G**) Cell lysates of HepG2 and MCF7 cells were analyzed by Western blot with specified antibodies. * *p* < 0.05, ** *p* < 0.01, *** *p* < 0.001, and **** *p* < 0.0001 represent the statistical significance compared to full (Ctrl) medium (**A**,**D**), to vector-transfected control cells (**B**,**E**), or to non-targeting control shRNA-transfected control cells (**C**,**F**).

Glutamine deprivation suppresses cell growth in several cancers [25]. Our results demonstrated that glutamine deprivation decreased HepG2 and MCF7 cell proliferation (Figure 6A,D). GS has been demonstrated to promote breast cancer, sarcoma, and glioblastoma cell survival under glutamine deprivation [11,12,13]. The overexpression of GS increased the proliferation of glutamine-deprived HepG2 cells (Figure 6B), whereas the survival-promoting effects were not observed in MCF7 cells overexpressing GS (Figure 6E). Furthermore, knockdown of GS decreased the proliferation of glutamine-deprived HepG2 cells at 24, 48, and 72 h (Figure 6C), whereas the cell growth of GS-silencing MCF7 cells following glutamine deprivation only significantly decreased at 72 h compared to non-targeting control shRNA-transfected control cells (Figure 6F). Compared to MCF7 cells, HepG2 cells expressed higher GS levels (Figure 6G). Studies have identified the overexpression of GS in hepatocellular carcinoma (HCC) and suggest that GS is a potential marker of HCC [26]. Consistent with this finding, our results indicate that liver cancer cells express high levels of GS that support cell survival under glutamine deprivation.

## 3. Discussion

Insulin plays a pleiotropic role in the regulation of nutrient uptake, gene expression, and synthesis of glycogen, proteins, DNA, and lipids [27]. However, whether insulin modulates glutamine metabolism is unknown. Our data reveal a novel finding: insulin increases GS expression, which is mediated by SREBP1. This finding indicates that insulin-induced SREBP1 not only promotes lipogenesis but also increases glutamine synthesis. Moreover, enhanced GS expression boosts glutamine-dependent anabolic pathways in nucleotide and protein synthesis [11] and also promotes lipogenesis and LD formation (as demonstrated in the current study), consistent with the functions of insulin. In addition to insulin, glutamine deprivation-induced GS expression is mediated by SREBP1, which further increases lipogenesis and supports cell survival under glutamine deprivation (Figure 5 and Figure 6). These findings reveal the expanded function of SREBP1 for enhancing glutamine synthesis along with lipogenesis, facilitating cancer cell growth.

Our results demonstrate that GS triggers a feedforward loop of O-GlcNAc-Sp1/SREBP1/ACC1 signaling that promotes lipogenesis. Moreover, GS mediates glutamine formation, contributing to the lipogenic acetyl-CoA production in the cytosol via oxidative glutamine metabolism through glutaminase, glutamate dehydrogenase, the citrate shuttle, and ATP citrate lyase [28,29]. On the other hand, glutamine is a carbon source for reductive glutamine metabolism in the production of citrate through cytoplasmic isocitrate dehydrogenase 1 (IDH1) or mitochondrial isocitrate dehydrogenase 2 (IDH2) and aconitase [30,31,32]. Notably, SREBP1 stimulates IDH1 expression that increases glutamine-derived lipogenesis [33]. These results support our finding that SREBP1 not only stimulates GS expression for the production of glutamine but also promotes glutamine-mediated lipogenesis through IDH1 in cancer.

Moreover, glutamine, glucose, and fatty acids are precursors of the hexosamine biosynthetic pathway (HBP) for the production of UDP-GlcNAc, a substrate of O-GlcNAc transferase. Increased protein O-GlcNAcylation on serine or threonine residues control protein stability, localization, transcriptional activity, and multiple other cellular functions [34,35]. This study indicates that GS promotes Sp1 O-GlcNAcylation, activating Sp1 to increase SREBP1/ACC1 expression and LD formation in liver and breast cancer cells. Similarly, the O-GlcNAcylation of Sp1 has been demonstrated to upregulate glycerol-3-phosphate acyltransferase 1 expression, which catalyzes lysophosphatidic acid production to protect cells in hypoxic conditions [36]. Moreover, the O-GlcNAcylation of fatty acid synthase increases protein stability, promoting lipogenesis and hepatic steatosis [37]. These findings suggest an important role of GS-mediated O-GlcNAcylation in the regulation of lipogenic enzymes to increase lipogenesis and cancer cell growth.

**Figure 7:**
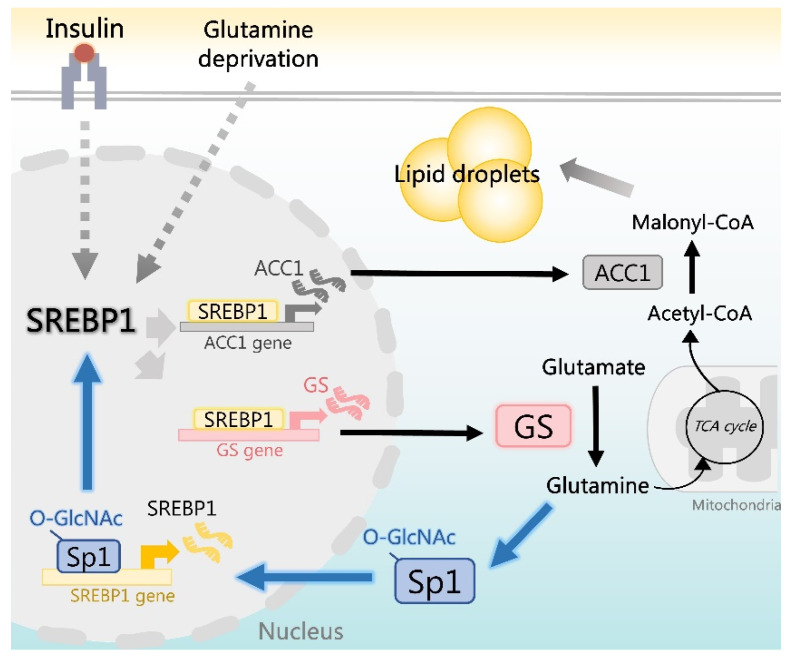
Schematic illustration of SREBP1-induced GS triggering a feedforward loop of O-GlcNAc-Sp1/SREBP1/ACC1 signaling that augments LD formation. SREBP1: sterol regulatory element-binding protein 1; GS: glutamine synthetase; O-GlcNAc: O-linked N-acetylglucosamine; Sp1: specificity protein 1; ACC1: acetyl-CoA carboxylase 1.

In conclusion, this study demonstrates that SREBP1 transcriptionally activates GS, promoting intracellular glutamine production and leading to increased O-GlcNAcylation of Sp1; in turn, O-GlcNAc-Sp1 transcriptionally upregulates the expression of SREBP1, resulting in a feedforward loop increasing lipogenesis and LD formation in liver and breast cancer cells (Figure 7). Hence, this study demonstrates the expanded function of SREBP1 for regulating GS expression and reveals a novel function of GS in the regulation of cancer lipogenesis.

## 4. Materials and Methods

### 4.1. Antibodies and Reagents

Antibodies used were as follows: SREBP1 (sc-13551, Santa Cruz Biotechnology, Dallas, TX, USA; 1:500 for WB), ACC (#3676, Cell Signaling Technology, Danvers, MA, USA; 1:1000 for WB), glyceraldehyde 3-phosphate dehydrogenase (GAPDH) (MA5-15738, ThermoFisher Scientific, Waltham, MA, USA; 1:10,000 for WB), GS (G2781, Sigma-Aldrich, Burlington, MA, USA; 1:1000–1:10,000 for WB, and GTX630654, GeneTex, Irvine, CA, USA; 1:1000–1:10,000 for WB), Sp1 (07-645, Millipore, Burlington, MA, USA; 1:1000 for WB), O-linked N-acetylglucosamine (clone RL2) (MABS157, Millipore, Burlington, MA, USA; 1:500 for WB), horseradish peroxidase (HRP)-conjugated anti-mouse IgG (AP124P, Millipore, Burlington, MA, USA; 1:500 for SREBP1; 1:10,000 for GAPDH; 1:500–1:5000 for GS from GeneTex; 1:500 for O-linked N-acetylglucosamine), HRP-conjugated anti-rabbit IgG (AP132P, Millipore, Burlington, MA, USA; 1:1000 for ACC; 1:500–1:5000 for GS from Sigma-Aldrich; 1:500 for Sp1), and normal mouse IgG (#31903, ThermoFisher Scientific, Waltham, MA, USA). Insulin (*Humulin R*) was purchased from Eli Lilly and Company. L-Methionine sulfoximine (MSO) (M5379, Sigma-Aldrich, Burlington, MA, USA) was purchased from Sigma-Aldrich. Betulin (sc-234016, Santa Cruz Biotechnology, Dallas, TX, USA) was purchased from Santa Cruz Biotechnology. The shRNA targeting GS and non-targeting control shRNA were purchased from Sigma-Aldrich.

### 4.2. Cell Lines and Culture

HepG2 and MCF7 cells were cultured in Dulbecco’s Modified Eagle Medium (DMEM) with 10% fetal bovine serum (FBS), 100 μg/mL streptomycin, and 100 units/mL penicillin as described previously [3]. The GS-expressing cells were established by retroviral infection of pBabe-GS construct and selected with puromycin (1 μg/mL) as a stable cell pool. The GS-silencing cells were established by lentiviral infection of the GS shRNA and selected with puromycin (1 μg/mL) for stable cell pools. Glutamine free DMEM (A1443001, Gibco, Waltham, MA, USA) and glutamine (CC515-0100, GeneDireX, Taichung, Taiwan) were purchased from Gibco and GeneDireX, respectively.

### 4.3. Western Blotting

Cells were harvested in a lysis buffer containing 10 mM Tris-HCl (pH 7.4), 100 mM NaCl, 1 mM EDTA, 0.1% SDS, 10% glycerol, 1% Triton X-100, 1 mM NaF, 0.5% sodium deoxycholate, 20 mM Na_4_P_2_O_7_, and 2 mM Na_3_VO_4_ supplemented with phenylmethylsulfonyl fluoride (PMSF) and protease inhibitors. The protein concentrations were determined by the Bio-Rad protein assay (Bio-Rad, Hercules, CA, USA). An equal amount of samples were analyzed by 10% sodium dodecyl sulfate-polyacrylamide gel electrophoresis (SDS-PAGE) and transferred to a polyvinylidene fluoride (PVDF) membrane. The membranes were immunoblotted with primary antibodies overnight at 4 °C followed by incubation with the HRP-conjugated secondary antibodies for 60 min at 25 °C. The immunoreactive signals were detected by using the enhanced chemiluminescence (ECL) detection kit (WBKLS0500, Millipore, Burlington, MA, USA) or the Trident Femto-ECL (GTX14698, GeneTex, Irvine, CA, USA). Images were visualized by a chemiluminescence imaging system (GeneGnome 5 chemiluminescent imaging system, Syngene, Frederick, MD, USA) or the Invitrogen iBright CL1000 Imaging Systems (ThermoFisher Scientific, Waltham, MA, USA). The relative amount of protein was quantified by densitometry analyses using ImageJ software (NIH).

### 4.4. Quantitative Reverse Transcription PCR (qRT-PCR)

Total cellular RNA was isolated with the TRIzol Reagent (Invitrogen, Waltham, MA, USA) and reverse transcribed with the high capacity cDNA Reverse Transcription Kit (Applied Biosystems, Waltham, MA, USA). Real-time quantitative PCR (qPCR) was performed with the Fast SYBR Green Master Mix (Applied Biosystems, Waltham, MA, USA) on the StepOnePlus Real-Time PCR system (Applied Biosystems, Waltham, MA, USA). The relative abundance of a particular mRNA was normalized to human GAPDH mRNA as an internal control. Specific primers are shown in Table S1.

### 4.5. Luciferase Assay

A 2528 bp DNA fragment comprising the GS promoter region and exon1 was PCR amplified from the human genomic DNA. The PCR product was cloned into the pGL4.17 vector containing the firefly luciferase reporter. To determine the promoter activity, cells were transiently transfected with vector, pcDNA3-nSREBP1a (nuclear form of SREBP1a encoding 485 AA) or pcDNA3-nSREBP1c (nuclear form of SREBP1c encoding 461 AA) [38] (50 ng) accompanied with the GS promoter constructs (50 ng) as well as the internal control plasmid pRL-CMV (5 ng) using jetPRIME^®^ (Polyplus transfection, Illkirch-Graffenstaden, France). Cells were seeded at 10^4^ cells per well in 96-well plates. Twenty-four hours post transfection, luciferase activities were determined following Dual-Glo^®^ Luciferase Assay System protocol (#E2920, Promega, Madison, WI, USA) with Infinite 200 PRO multimode plate reader (Tecan, Männedorf, Switzerland). The ratios of firefly luminescence versus Renilla luminescence were calculated as promoter activities and the promoter activities of nSREBP1a or nSREBP1c-transfected cells were normalized to that of vector-transfected control cells as relative promoter activities.

### 4.6. Intracellular Glutamine Measurement

Cells were seeded at 5 × 10^5^ cells per well in 60-mm dish followed by 200 nM insulin treatment for 72 h. The collected cells were separated equally to measure intracellular glutamine levels and protein concentrations. Glutamine levels were measured by glutamine colorimetric assay kit (#K556, Biovision, Milpitas, CA, USA) according to the manufacturer’s instructions. Glutamine concentration is expressed as nmol/mg of protein.

### 4.7. ChIP Assay

Chromatin immunoprecipitation (ChIP) assays were performed as described previously [3]. Briefly, proteins were cross-linked to DNA for 10 min with the addition of 1% formaldehyde and the reaction was stopped by adding 125 mM glycine. Cells were washed and scraped with phosphate buffer saline (PBS) containing protease inhibitors. Cells were collected and lysed with the ChIP lysis buffer (50 mM HEPES-KOH pH 7.5, 1 mM EDTA pH 8.0, 140 mM NaCl, 0.1% SDS, 0.1% sodium deoxycholate, 1% Triton X-100, and protease inhibitors). Samples were sonicated with the sonicator (Bioruptor^TM^ UCD-200, Diagenode, Denville, NJ, USA) and target proteins were immunoprecipitated with the Dynabeads™ Protein G (#10004D, Invitrogen, Waltham, MA, USA) in the dilution buffer with SREBP1 or normal mouse IgG antibodies overnight at 4 °C. The beads were washed with the wash buffer, the final wash buffer, and finally eluted in the elution buffer (100 mM NaHCO_3_, 1% SDS, and proteinase K) with shaking at 55 °C for 2 h and inactivating proteinase K by incubation at 95 °C for 10 min. The DNA cleanup kit (D4034, Zymo Research, Irvine, CA, USA) was used to purify all DNA samples before qPCR analysis. Specific primers for ChIP are shown in Table S1.

### 4.8. Lipid Staining

For lipid staining, Oil Red O staining was performed according to manufacturer’s instructions (O0625, Sigma-Aldrich, Burlington, MA, USA). Briefly, cells were washed once with PBS and fixed in 4% paraformaldehyde overnight. Cells were washed once with 60% isopropanol and incubated with Oil Red O solution for 10 min. Cells were then washed 5 times with ddH_2_O and photographed using the bright-field microscopy. Oil Red O were eluted by adding 100% isopropanol for 2 h, and the absorbance was measured at 500 nm and normalized to its protein concentration. BODIPY 505/515 staining was also performed according to manufacturer’s instructions (#D3921, Invitrogen, Waltham, MA, USA). Briefly, cells were washed once with PBS and fixed in 4% paraformaldehyde for 15 min. Cells were washed three times with PBS and incubated with 0.2 μM BODIPY 505/515 for 30 min. The cells were then counterstained with 1.5 μg/mL DAPI (H-1200, Vector Laboratories, Burlingame, CA, USA) for 5 min. Cells were examined and photographed using the fluorescence microscopy.

### 4.9. Cell Growth Assay

Cells (10^4^/well) were seeded in 24-well culture plates for 24 h, and then treated with various treatments. The 3-(4,5-dimethylthiazol-2-yl)-2,5-diphenyl tetrazolium bromide (MTT) assay was performed to measure cell growth rate. Briefly, cells were washed with PBS and then incubated with the MTT solution (1 mg/mL) at 37 °C for 4–6 h. After removing the MTT solution, dimethyl sulfoxide (DMSO) was added to dissolve the purple formazan crystals, and the absorbance was measured at 570 nm with a reference wavelength of 630 nm using the VersaMax Microplate Reader (Molecular Devices, San Jose, CA, USA).

### 4.10. Database Analyses

For SREBP1 and GS expression in liver and breast cancer, a public dataset containing normal and liver cancer specimens (GSE87630) or normal and breast cancer specimens (GSE83591) was respectively analyzed from GEO datasets (https://www.ncbi.nlm.nih.gov/gds/, accessed on 27 September 2019) via analysis platform of GEO2R. Next, analysis of expression correlation between SREBP1 and GS in TCGA dataset of breast cancer was analyzed by using UCSC Xena platform (https://xena.ucsc.edu/, accessed on 27 September 2019).

### 4.11. Statistical Analyses

Unless indicated, results are expressed as mean plus standard error of the mean (SEM) from at least three independent experiments. Experiments comparing two groups were analyzed by the two-tailed unpaired Student’s *t*-test with GraphPad Prism 5 for Windows (GraphPad Software Inc, San Diego, CA, USA). Differences among multiple groups were analyzed by ANOVA followed by the Bonferroni or Dunnett post hoc test. Correlation analysis was evaluated by Pearson’s correlation (with a two-tailed test of significance) with GraphPad Prism 5. The statistical significance level was 0.05. * *p* < 0.05, ** *p* < 0.01 and *** *p* < 0.001 or ^#^ *p* < 0.05, ^##^ *p* < 0.01 and ^###^ *p* < 0.001 were considered to be statistically significant.

## Data Availability

All data are contained within this manuscript.

## Supplementary Materials

The following are available online at https://www.mdpi.com/article/10.3390/ijms22189814/s1.

## Abbreviations

ACC1	acetyl-CoA carboxylase 1
ChIP	chromatin immunoprecipitation
DMEM	Dulbecco’s Modified Eagle Medium
DMSO	dimethyl sulfoxide
ECL	enhanced chemiluminescence
FBS	fetal bovine serum
GS	glutamine synthetase
HBP	hexosamine biosynthetic pathway
HCC	hepatocellular carcinoma
IDH1	isocitrate dehydrogenase 1
IDH2	isocitrate dehydrogenase 2
LD	lipid droplet
MTT	3-(4,5-dimethylthiazol-2-yl)-2,5-diphenyl tetrazolium bromide
O-GlcNAcylation	O-linked N-acetylglucosaminylation
PMSF	phenylmethylsulfonyl fluoride
PVDF	polyvinylidene fluoride
PBS	phosphate buffer saline
qPCR	quantitative PCR
SREBP1	sterol regulatory element-binding protein 1
Sp1	specificity protein 1
SRE	sterol regulatory element
SDS-PAGE	sodium dodecyl sulfate-polyacrylamide gel electrophoresis
SEM	standard error of the mean
UDP-GlcNAc	uridine-diphosphate-N-acetylglucosamine
