# Supplementary material for: SREBP1-Induced Glutamine Synthetase Triggers a Feedforward Loop to Upregulate SREBP1 through Sp1 O-GlcNAcylation and Augments Lipid Droplet Formation in Cancer Cells
Source: Int J Mol Sci. 2021 Sep 10;22(18):9814. doi: 10.3390/ijms22189814 (PMC8469118; doi:10.3390/ijms22189814)
Supplement: Supplementary file 1 [file ijms-22-09814-s001.zip › ijms-1313800-supplementary.pdf]

Supplementary Information

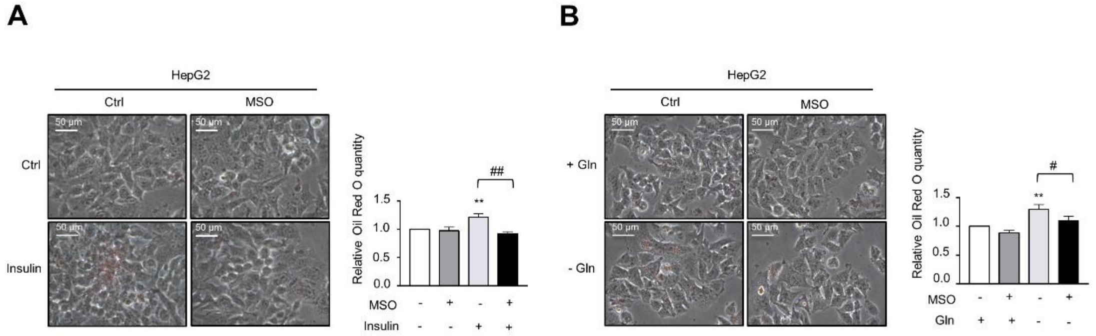

Figure S1. Inhibition of GS decreases insulin or glutamine deprivation-induced LD formation. (**A**) HepG2 cells were treated with 200 μM GS inhibitor MSO for 30 min prior to 200 nM insulin treatment for 48 h. (**B**) HepG2 cells were treated with 200 μM GS inhibitor MSO for 30 min prior to glutamine deprivation (- Gln) for 24 h. LD was stained with Oil Red O and normalized to protein concentrations for the quantitative analysis. Data represent means plus SEM. ** $p <$ 0.01 compared to control cells. $^{\#} p < 0.05$ and $^{\#\#} p < 0.01$ between indicated groups.

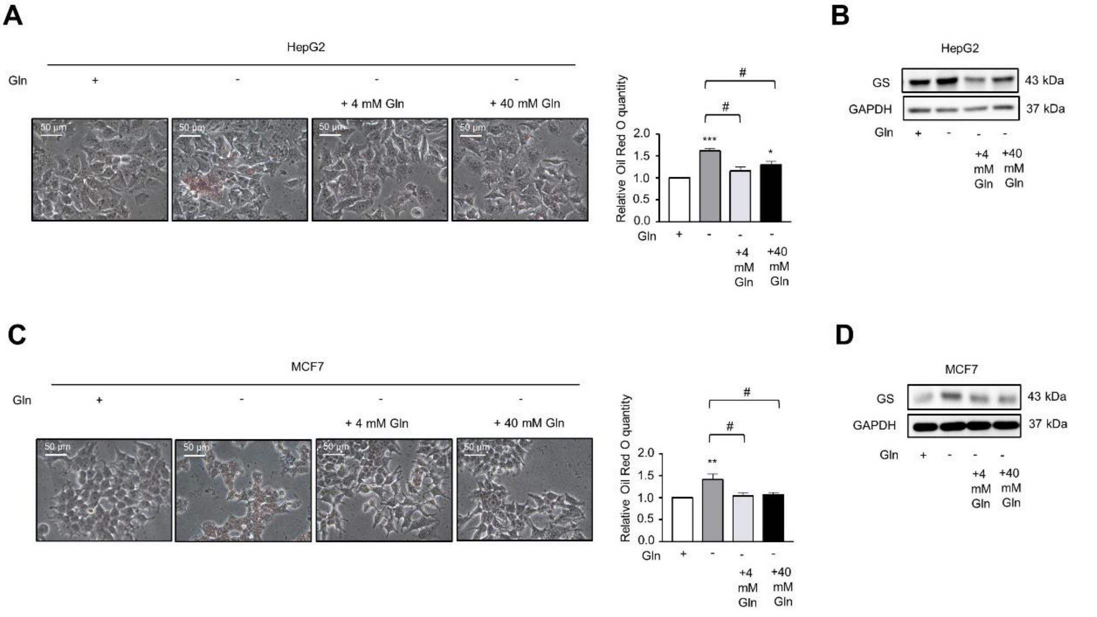

Figure S2. The addition of glutamine decreases glutamine deprivation-induced LD formation and GS expression.

(**A** and **B**) HepG2 cells were cultured under glutamine deprivation for 24 h and then added with 4 mM Gln or 40 mM Gln for 24 h. (**C** and **D**) MCF7 cells were cultured under glutamine deprivation for 16 h and then added with 4 mM Gln or 40 mM Gln for 8 h. (**A** and **C**) LD was stained with Oil Red O and normalized to protein concentrations for the quantitative analysis. (**B** and **D**) Cell lysates were analyzed by Western blot with specified antibodies. Data represent means plus SEM. * $p < 0.05$, ** $p < 0.01$, and *** $p < 0.001$ compared to control cells. $^{\#} p < 0.05$ between indicated groups.

Table S1. Primer list

qRT-PCR primers

Gene	Primer sequence (5′-3′)
ACC1	(F) CCCAGATTCTGCGTTTAAGA
	(R) CATCCACAATGTAAGCACCA
SREBP1	(F) CCATGGATTGCACTTTCGAA
	(R) GGCCAGGGAAGTCACTGTCTT
GS	(F) ACTTCGCAAGCGGCACCA
	(R) TTGGCTACACCAGCAGAAAA
GAPDH	(F) AAGGTCGGAGTCAACGGATTTG
	(R) CCATGGGTGGAATCATATTGGAA

ChIP primers

Gene	Primer sequence (5′-3′)
GS	(F) CAAAAGTCGCTATGGGGAAAG
	(R) GCCACATGAAGCCTGTTTCT
